# Corilagin prevents non-alcoholic fatty liver disease *via* improving lipid metabolism and glucose homeostasis in high fat diet-fed mice

**DOI:** 10.3389/fnut.2022.983450

**Published:** 2022-08-17

**Authors:** Mingjuan Liao, Rong Zhang, Yongling Wang, Ziming Mao, Jing Wu, Huaqi Guo, Kaiwen Zhang, Yu Jing, Caoxu Zhang, Huaidong Song, Xia Chen, Gang Wei

**Affiliations:** ^1^Department of Traditional Chinese Medicine, The Ninth People’s Hospital, Shanghai Jiao Tong University School of Medicine, Shanghai, China; ^2^Department of Nephrology, Shanghai General Hospital, Shanghai Jiao Tong University School of Medicine, Shanghai, China; ^3^The Core Laboratory in Medical Center of Clinical Research, Department of Molecular Diagnostics & Endocrinology, Shanghai Ninth People’s Hospital, State Key Laboratory of Medical Genomics, Shanghai Jiao Tong University School of Medicine, Shanghai, China; ^4^Department of Endocrinology, Shanghai Gongli Hospital, Shanghai, China; ^5^Key Laboratory of Pollution Exposure and Health Intervention of Zhejiang Province, Hangzhou, China; ^6^Beijing Key Laboratory of Diabetes Research and Care, Department of Endocrinology, Beijing Diabetes Institute, Beijing Tongren Hospital, Capital Medical University, Beijing, China

**Keywords:** NAFLD activity score (NAS), hepatic lipid deposition, insulin resistance, RNA-seq analysis, corilagin

## Abstract

Non-alcoholic fatty liver disease (NAFLD) has been considered to be one of the most common chronic liver diseases. However, no validated pharmacological therapies have been officially proved in clinic due to its complex pathogenesis. The purpose of this study was to examine the protective effects of Corilagin (referred to Cori) against NAFLD in mice under a high fat diet (HFD) condition. Mice were fed either a normal control diet (NCD) or HFD with or without Cori (5 or 10 mg/kg body weight) for 15 weeks. In our results, Cori treatment significantly attenuated HFD-induced hepatic steatosis, high NAFLD activity score (NAD) and liver injury. Consistently, Cori treatment remarkably alleviated HFD-induced hepatic lipid accumulation (e.g., triglycerides (TG) and total cholesterol (TC) contents in liver), and improved plasma lipid concentrations (e.g., plasma TG, TC, low-density lipoprotein cholesterol (LDL-c), high-density lipoprotein cholesterol (HDL-c)). Moreover, Cori treatment ameliorated NAFLD associated metabolic disorders such as glucose intolerance and insulin resistance in HFD-fed mice. Additionally, Cori treatment dramatically changed HFD-induced liver gene expression profiles, and identified overlapped differentially expressed genes (DEGs) between NCD vs. HFD group and HFD vs. HCR (high fat diet plus treatment with Cori) group. With these DEGs, we observed a marked enrichment of Gene Ontology (GO) terms and Kyoto Encyclopedia of Genes and Genomes (KEGG) pathways, which were closely associated with the metabolic balance in liver. Particularly, we found several potential hub proteins against NAFLD development with analyses of protein-protein interaction (PPI) network and qPCR assays. Collectively, our results revealed the important protective effects of Cori against the progress of NAFLD, which was probably mediated through improving dysregulated lipid metabolism and insulin resistance in HFD-fed mice. Additionally, Cori-dependent overlapped DEGs might serve as a featured NAFLD-associated gene expression signature for the diagnosis, treatment, as well as drug discovery and development of NAFLD in the near future.

## Introduction

Non-alcoholic fatty liver disease (NAFLD) is an emerging metabolic disorder around the world, which is featured by excessive hepatic lipid deposition without preexisting liver diseases caused by alcohol abuse (1). Epidemiological surveys show that the annual prevalence of NAFLD has been increasing due to an oversupplied lifestyle, which reaches about 25% in adult globally (2, 3). As the most prevalent chronic liver disorder, NAFLD can further result in severe liver pathologies, such as fibrosis, cirrhosis and hepatocellular carcinoma (HCC), resulting in an increase in the risks for overall mortality (4). Importantly, with the interventions of lifestyle or nutrition modifications, the progress of NAFLD is potentially reversible at an early stage (5). However, pharmacological intervention is needed when the disease is serious. More alarmingly, no effective pharmacological therapies for its treatment have been officially approved in clinic yet, although several drugs are under evaluation (6). Therefore, there is an urgent necessity to develop the novel medical interventions for the treatment of NAFLD.

Recently, natural products play a pivotal role in drug discovery, which also provide new clues to molecular mechanisms of diseases (7, 8). A number of studies have demonstrated that natural compounds (e.g., indirubin, rutin) exert protective roles against the hepatic steatosis *in vitro* and *in vivo* (9, 10). Among these, Corilagin (referred subsequentlyto as Cori, β-1-O-galloyl-3, 6-(R)-hexahydroxydiphenoyl-D-glucose), a polyphenols tanic acid chemical, exists in several herbaceous plants, including *Dimocarpus longana*, *Phyllanthus emblica*, *Phyllanthus reticulatus*, and *Geranium wilfordii* (11–13). Of note, Cori displays multiple bioactive properties, including anti-tumoral, anti-oxidative, anti-inflammatory, anti-viral and hepatoprotective activities. Liao et al. and Feng et al. reported that Cori prevented acetaminophen-induced hepatotoxicity by inhibiting NF-κB and ERK/JK/MAPK signaling pathways and by stimulating the AMPK/GSK3β-Nrf2 signaling pathway, respectively (14, 15). Likewise, Zhou et al. and Li et al. demonstrated that Cori attenuated CCL4- and schistosome egg-induced liver fibrosis through blocking mir-21-mediated TGF-β1/Smad signaling pathway and through inhibiting M2 macrophage polarization via suppressing IL-13Rα1 signaling pathway, respectively (16, 17). Moreover, Yang et al. showed that Cori ameliorated α-naphthylissthipcyanate-induced intrahepatic cholestasis by regulating FXR-associated pathways (18). Additionally, Li et al. revealed that Cori exerted obvious therapeutic efficacy for the treatment of cholangiocarcinoma *in vivo* and *in vitro* by inhibiting Notch signal pathway (19).

The pathogenesis of NAFLD is complex, and multiple mechanisms may contribute to hepatic lipid deposition (20). In particular, our group previously revealed that Cori suppressed palmitic acid/oleic acid (PA/OA, lipids) -induced hepatic lipid deposition in AML12 cell line, which was associated with the improvement of metabolism adaption, including the diminished oxidative stress, restored autophagic flux, as well as improved mitochondrial function (11). Nevertheless, the protective roles and molecular mechanisms of Cori against NAFLD development largely remains to be elucidated. Therefore, the purpose of this study was to evaluate the protective effect of Cori against high fat diet (HFD)- induced NAFLD in mice.

## Materials and methods

### Animals and treatments

Male C57BL/6 mice (four-week-old) were purchased from Beijing SPF experimental animal Technology Co., Ltd. (Certificate number: SCXK2019-0010, Beijing, China). These mice were maintained under a standard environment (diet and filtered water *ad libitum*, 50 ± 5% humidity, 20 ± 2°C, 12:12-h light/dark cycle). All the experiments were approved by the ethics committee of the Medical School of Shanghai Jiao Tong University, Shanghai, China. After one-week acclimatization, the mice were then randomly separated into four groups (*n* = 8 per group) under indicated treatments for 15 weeks: (i) mice fed with normal control diet (NCD, D12450B, Research Diet); (ii) mice fed with high fat diet (HFD, D12492, Research Diet); (iii) mice fed with HFD and simultaneously treated with Cori (intraperitoneal injection, interval for 48 h) at a dose of 5 mg/kg/day (HCR-L); (iv) mice fed with HFD and simultaneously treated with Cori (intraperitoneal injection, interval for 48 h) at a dose of 10 mg/kg/day (HCR-H). Cori was obtained from BioPurify Phytochemicals Ltd. (Cat no. BP0393, Chengdu, China). Mice in NCD and HFD group were given corresponding vehicle as control. Body weight and food intake in indicated groups were determined every week. The index of Liver or epididymal fat index was calculated as the ratio of liver or epididymal fat weight to body weight, respectively. Following fasting overnight at the end of experiments, mice were sacrificed after anesthetized, and their blood samples were collected from the orbital venous plexus. Fresh liver and eppididymal fat were removed, weighted, fixed in 4% formaldehyde (Elabscience, China) in a 1.5 ml Eppendorf tube (NEST Biotechnology, China) or snap-frozen in liquid nitrogen and stored at –80°C for further research.

### Histological evaluation

For liver pathological changes observation, the liver and epididymal adipose tissue in indicated groups were fixed with 4% paraformaldehyde, embedded, sectioned and cut into thin slices (5-μm thickness). The slices were further stained hematoxylin and eosin (H&E staining) and observed with a light microscope (Zeiss, Germany). Based on the results of H&E staining, NAFLD activity score (NAS) was evaluated as previously described (21). For hepatic lipid deposition analysis, the liver tissues were embedded in OCT-freeze medium, and cut into thin slices (12-μm thickness) (Procell Life Science & Technology Co., Ltd.). The slices were then stained with the work solution of Oil Red O (Oil Red O staining) (abs7049, Absin Bioscience, Inc., Shanghai, China). Images were observed and captured with a light microscope (Zeiss, Germany).

### Hepatic and plasma biochemical analysis

Hepatic lipids such as triglycerides (TG) and total cholesterol (TC) were determined by commercial kits (Applygen Beijing, China and Solarbio, Beijing, China, respectively) according to manufacturer’s protocols. Briefly, the blood samples from indicated groups were kept for 30 min at room temperature, followed by centrifugation (3000 rpm/min) for 15 min at 4°C. The serum parameters of liver function such as alanine aminotransferase (ALT), aspartate aminotransferase (AST), and plasma lipid concentration such as low-density lipoprotein cholesterol (LDL-c), high-density lipoprotein cholesterol (HDL-c), triglycerides (TG) and total cholesterol (TC) were measured with an automatic chemistry analyzer (Hitachi Ltd., Japan). Plasma fatty acid binding protein (FABP4) and free fat acids (FFAs) were examined by using commercial ELISA Kits (Cusabio Biotech., Wuhan, China; Wako chem., Osaka, Japan, respectively).

### Glucose tolerant test (GTT) and insulin tolerant test (ITT)

For glucose and insulin tolerance test, mice were fasted for 6 h with free access to water. The mice were intraperitoneally (i.p.) injected with D-glucose (1.2 g/kg body weight, Sigma, United States) or insulin (0.7 IU/kg body weight, Novolin, United States), respectively. Then, the blood glucose level of each mouse was recorded at 0, 15, 30, 60, 90, and 120 min using a glucose monitor (Accu-Chek, Roche, United States). The insulin resistant parameters were evaluated with homeostatic model assessment of insulin resistance (HOMA-IR), which were calculated with the calculator of HOMA2 model as previously descripted (22).

### RNA sequencing (RNA-Seq) and data analysis

The RNA-Seq for liver samples from indicated groups were conducted in Personalbio technology Co. Ltd. (Shanghai, China). Briefly, Total RNA were extracted from liver tissues in indicated groups using TRIzol reagent (Invitrogen, United States). Then, RNAs were quantified (NanoDrop ND-2000), qualified (agarose gel electrophoresis), cDNA synthesized (Illumina kits), RNA-Seq libraries prepared and qualified (Agilent 2100 Bioanalyzer and qPCR method). RNA-Seq was subsequently performed on the Illumina HiSeq 2000 sequencing platform. Raw reads stored with FASTQ format were obtained from indicated groups. After filtering the low-quality data or data containing adapters, clean reads were mapped to the reference genome using HISAT2 software. Fragments Per Kilobase of exon model per Million mapped fragments (FPKM) was used to determine the gene expression abundance, and DESeq2 was used to further analyze these differential expression genes (DEGs) in indicated groups, respectively. Scatter plots, volcano lots, hierarchical clustering (Cluter software), Venn diagram, Gene Ontology (GO), Kyoto Encyclopedia of Genes and Genomes (KEGG) pathway analysis (DAVID database) and protein-protein interaction (PPI) network (Cytoscape software) were performed and graphed for bioinformatics analysis of differentially expressed genes (DEGs) in indicated groups.

### Quantitative real-time PCR (qPCR) assay

Total RNA extracted from liver tissues in indicated groups by using Fast Pure Cell/Tiss Total RNA isolation kit V2 (Vazyme Biotech, Nanjing, China). 500 ng of total RNAs in each sample were used to conduct reverse transcriptions with Q RT SuperMix kit of Vazyme HiScript II. qPCR analysis were performed by using qPCR SYBR Green Master Mix of Vazyme AceQ on a QuantStudio 6 Flex Real-Time PCR System (ABI). 18S was used as a control and the sequences of gene-specific primers are listed in Table 1.

**TABLE 1:** Sequence of primers used for qPCR.

Gene	Forward primer (5′-3′)	Reverse primer (5′-3′)
Hsd3b5	AGTGCTAAATAGCGTGTTTACCA	ACTTTTTGTGTAGTGTCTCCCTG
Cyp2c40	GGCTCACAGCCTATTGTGGTA	TCAAAAACCGGAATCCTTCCTC
Cspg5	AGGAACCTCAGAAAATCACCCT	TGGTGGGGTAGAAATCAGACTC
Cyp7a1	GGGATTGCTGTGGTAGTGAGC	GGTATGGAATCAACCCGTTGTC
Cyp17a1	GTCGCCTTTGCGGATAGTAGT	TGAGTTGGCTTCCTGACATATCA
Cyp2c38	CACGGCCCATTGTTGTATTGC	TGAACCGTCTTGTCTCTTTCCA
Cyp2b9	GCTCATTCTCTGGTCAGATGTTT	CGCTTGTGGTCTCAGTTCCA
Erbb4	TCCCCCAGGCTTTCAACATAC	GCACCCTGAGCTACTGGAG
Gna14	AGTGGGAAAAGCACCTTTATCAA	TCCCTGATGATCTGGGCATTT
Chrm1	AGTCCCAACATCACCGTCTTG	CAGGTTGCCTGTCACTGTAGC
Gpc1	GGAGAGCGCACTCCATGAC	CTCAGCATATAGGTCCCGGAA
Egfr	GCATCATGGGAGAGAACAACA	CTGCCATTGAACGTACCCAGA
Cd36	AGATGACGTGGCAAAGAACAG	CCTTGGCTAGATAACGAACTCTG
18S	ACCTGGTTGATCCTGCCAGTAG	TTAATGAGCCATTCGCAGTTTC

### Statistical analysis

All data were indicated as mean ± SD, and analyzed using GraphPad Prism7.0 and Microsoft Excel. The statistical significance of differences was evaluated using the Student’s *t*-test (2-tailed), One-way analysis of variance (ANOVA), or two-way ANOVA as indicated. *p* value of less than 0.05 was considered to be a statistically significant difference.

## Results

### Cori attenuated HFD-induced high NAFLD activity score (NAS) and liver injury

**FIGURE 1:**
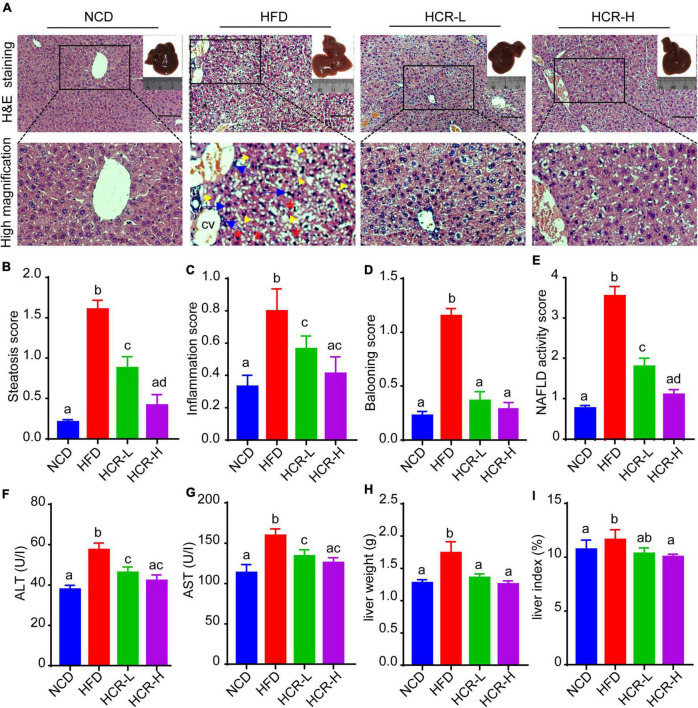
Cori reduced the high NAFLD activity score (NAS) and inhibited liver damage in HFD-fed mice. **(A)** H&E staining of livers in indicated groups. As shown in high magnification image of HFD group, arrowhead (yellow color) indicates steatosis; arrow (blue color) indicates inflammatory cells infiltration; asterisk (red color) indicates a balloon-like change; cv indicates central vein. Bar = 50 μm. Liver gross morphology of indicated group was represented in the upper right corner. **(B)** Hepatic steatosis score, **(C)** inflammation score, **(D)** ballooning score, and **(E)** NAFLD activity score (NAS) were quantified from indicated groups. **(F)** Serum ALT and **(G)** AST levels were measured by biochemical analysis. **(H)** Liver weights of indicated groups. **(I)** Liver index (the ratio of liver weight to body weight) in indicated groups. NCD, normal chow diet; HFD, high fat diet; HCR-L, high fat diet plus treatment with low dose of Cori (5 mg/kg/2 days); HCR-H, high fat diet plus treatment with high dose of Cori (10 mg/kg/2 days). Values were means ± SD, and for statistical analysis, one-way ANOVA were performed between indicated groups.

To investigate the beneficial effects of Cori (CAS: 23094-69-1, Supplementary Figure 1A) on HFD-induced pathological process of NAFLD, we collected the liver tissues at the end of experiments and detected the severity grade of hepatic steatosis and NAFLD activity score (NAS) by hematoxylin and eosin (H&E) staining of the liver tissue samples. As shown in Figure 1A, we found that the liver gross morphology of the HFD group was obviously pale and enlarged compared to the normal control diet (NCD) mice, whereas the appearance of the livers in Cori-treated group was rescued to almost normalshape. Notably, we did not observed marked abdominal adhesions and peritionitis in mice. Moreover, pathophysiological examination revealed that the lipid accumulation was obvious in livers of mice fed on a HFD compared to the normal control diet (NCD) mice, whereas the number and size of lipid droplets were reduced after treating with Cori under HFD conditions, as evidenced by fewer vacuoles in H&E stained liver sections. Consistently, the steatosis score, inflammation score and ballooning score in mice on HFD feeding were significantly higher than NCD group, respectively (Figures 1B–D); However, Cori obviously reduced the high histological scores, indicating an improvement in the grade of hepatic steatosis, inflammation and ballooning degeneration (Figures 1B–D). As a result, in mice on HFD-induced NAFLD model, Cori treatment strikingly lowered total NAFLD activity score (NAS) in a dose-dependent manner (decreased by 1.75 in HCR-L group and 2.44 in HCR-H group, respectively), which is the sum of hepatic steatosis inflammation and ballooning scores, in a dose- dependent manner (Figure 1E). Moreover, the plasma ALT and AST levels, two common biomakers of hepatocellular injury, were significantly increased in HFD-induced mice, as compared to NCD mice, but the levels were both attenuated after Cori administration (Figures 1F,G). Additionally, we observed an inhibited body weight gain after treatment with Cori compared with HFD-fed only mice, although no differences in food consumption among mice in HFD, HCR-L and HCR-H group. (Supplementary Figures 1B,C). Interestingly, the liver weight and liver index (%, liver weight ratios) were dramatically increased in HFD-fed mice relative to NCD group (4.65% vs. 4.05%), whereas Cori treatment resulted in a significant reduction in their absolute liver weights (decreased by 0.38 in HCR-L group and 0.48 g in HCR-H group, respectively) and relative liver weights (decreased by 0.53% in HCR-L group and 0.64% in HCR-H group, respectively) in comparison to HFD-fed only group (Figures 1H,I). These results indicated that Cori inhibited the pathological development of HFD-triggered NAFLD by reducing the higher NAFLD activity score (NAS) and preventing the liver injury.

### Cori improved HFD-induced hepatic lipid deposition and plasma lipid concentration

**FIGURE 2:**
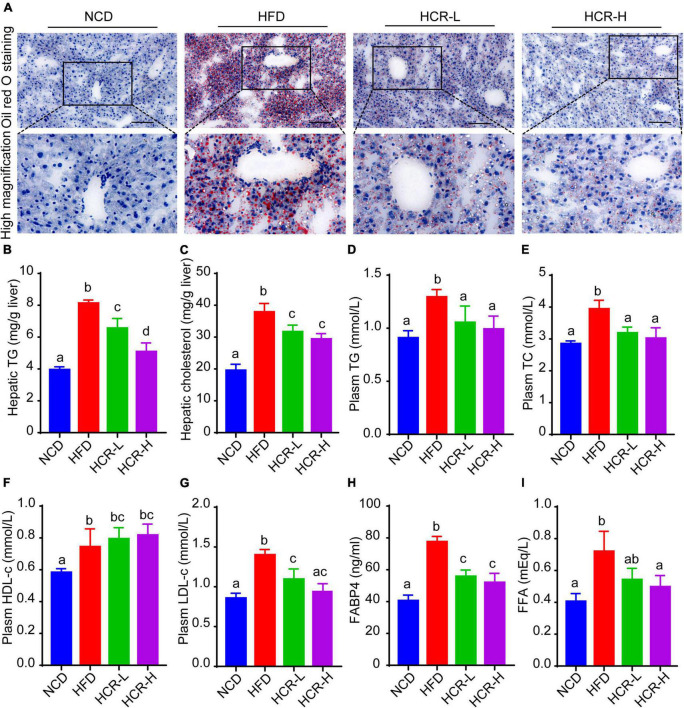
Cori alleviated hepatic and plasma lipid concentrations in HFD-fed mice. **(A)** Oil Red O staining of liver in indicated groups. Bar = 50 μm. **(B)** TG and **(C)** TC levels in liver were detected. **(D)** Plasm TG, **(E)** TC, **(F)** HDL-c, **(G)** LDH-c were tested. **(H)** FABP4 in plasma was measured. **(I)** FFA in plasma was examined. Values were means ± SD, and for statistical analysis, one-way ANOVA were performed between indicated groups.

To further confirm the protective role of Cori in HFD- mediated hepatic lipid accumulation, we determined liver total lipid contents *via* liver Oil Red O staining. Consistent with the H&E results, Oil Red Staining demonstrated that the livers of HFD-fed mice exhibited numerous red-stained lipid globules compare to NCD mice (Figure 2A and Supplementary Figure 1D). Notably, the lipid droplets intheliversofHFD model mice were of varying size, which clearly showed that HFD feeding resulted in hepatic lipid accumulation, exhibiting mixed micro-vesicular to macro-vesicular steatosis. Interestingly, Cori treatment substantially suppressed intrahepatic ectopic lipid deposition of mice with HFD feeding, as demonstrated by fewer red-stained lipid droplets and by quantitative analysis of the lipid positive area in their frozen liver sections (Figure 2A and Supplementary Figure 1D). More importantly, liver TG and TC contents were markedly higher in HFD mice than NCD mice, whereas Cori treatment dramatically reversed the HFD-induced TG and TC accumulation (Figures 2B,C). Additionally, by treating with Cori, we observed a marked reduction of epididymal fat weight (decreased by 1.05 g in HCR-L group and 1.92 g in HCR-H group, respectively) as well as epididymal fat index (%) (decreased by 1.88% in HCR-L group and 4.35% in HCR-H group, respectively) in HFD-fed mice (Supplementary Figure 1E), which might contribute to the decreased levels of FFAs and FABP4 in plasma. These results further confirmed that Cori could prevent HFD-induced hepatic lipid accumulation.

Hyperlipidemia, a typical feature in NAFLD conditions, is closely associated with the pathological development of hepatic steatosis (23). As shown in Figures 2D–G, biochemical analysis revealed that plasma levels of TG, TC, and LDH-c in HFD group were significantly increased, compared to NCD group. By contrast, Cori treatment abrogated the changes of plasma lipid concentrations caused by HFD. In addition, compared with the NCD group, plasm HDL-c levels were slightly but no significantly increased in HFD-fed mice, whereas Cori treatment further increased the HDL-c concentrations in plasma, leading to a statistically significant difference relative to NCD mice. Moreover, plasma fatty acid (FFA) and FFA-binding protein 4 (FABP4) were significantly elevated in HFD-fed mice, whereas Cori treatment remarkably alleviated the increase of plasm FFA and FABP4 contents under HFD conditions. These results indicated that Cori could inhibit HFD-induced hepatic lipid accumulation and improve plasma lipid concentrations, thus blocking the pathological process of NAFLD.

### Cori ameliorated HFD-induced glucose intolerance and insulin resistance

**FIGURE 3:**
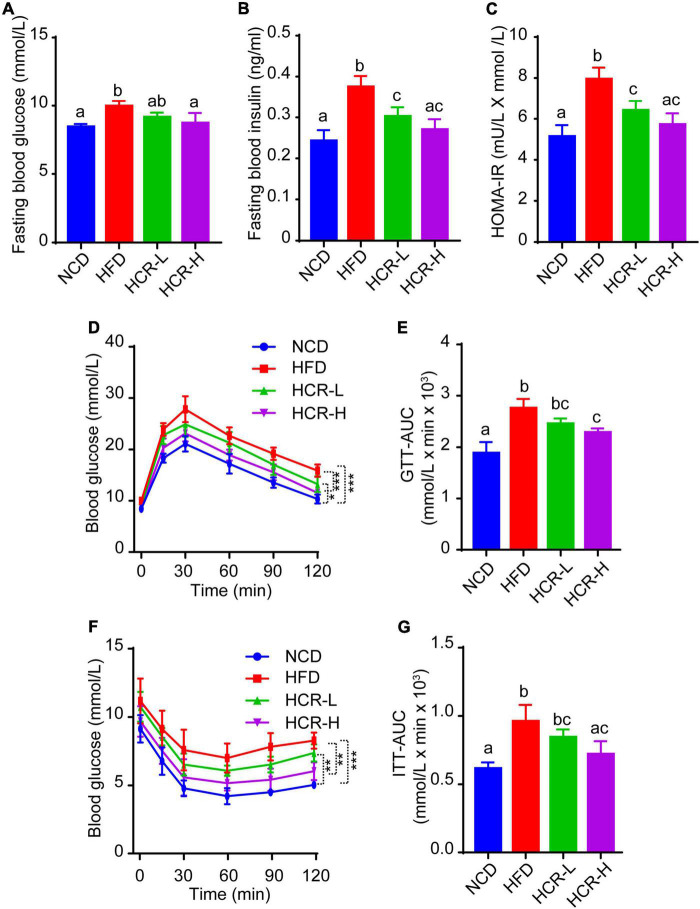
Cori improved glucose intolerance and insulin resistance in HFD-fed mice. **(A)** Levels of fasting blood glucose, and **(B)** levels of fasting insulin were detected in indicated groups. **(C)** HOMA-IR was calculated with the formula: [fasting glucose (mU/L)* fasting insulin (mmol/L)]/22.5. **(D)** Glucose tolerance test (GTT) was examined, and **(E)** the area under the curve (AUC) for GTT was calculated. **(F)** Insulin tolerance test (ITT) was tested, and **(G)** the area under the curve (AUC) for ITT was calculated.

As insulin resistance (IR) caused by HFD feeding is a crucial factor that affects the histological severity of NAFLD, we next considered in the analysis of the status of IR with parameters such as fasting blood glucose levels, fasting insulin levels, and homeostasis model assessment of insulin resistance (HOMA-IR) value. As shown in Figures 3A–C, when mice fed on a HFD, fasting blood glucose and insulin levels both were significantly increased, compared to NCD mice, thereby resulting in a higher HOMA-IR value (7.95 vs 5.14). Conversely, Cori treatment remarkably inhibited HFD-induced increase of fasting blood glucose and insulin concentrations, and strongly reversed the HOMA-IR value (decreased by 1.54 in HCR-L group and 2.23 in HCR-H group) (Figures 3A–C), exerting its ameliorating effects against insulin resistance.

To further investigate whether Cori treatment has an improvement effect on glucose intolerance and insulin resistance during the progressive development of NAFLD, we performed glucose tolerance test (GTT) and insulin tolerance test (ITT) assays. As shown in Figures 3D–G, comparing with NCD group, HFD feeding caused obvious glucose intolerance and insulin resistance in mice after glucose and insulin load (i.p.) (Figures 3D–G), and resulted in higher values of area under the curve (AUC) for GTT and ITT, respectively (Figures 3E,G). Interestingly, Cori treatment substantially improved their glucose disposal, as manifested by GTT and ITT assays and quantification of AUC values in mice under HFD conditions (Figures 3D–G). Taken together, these results indicated Cori treatment reversedHFD feeding-induced glucose intolerance and insulin resistance, which was also associated with NAFLD progression.

### Cori altered HFD-induced gene expression profiles in live tissue

**FIGURE 4:**
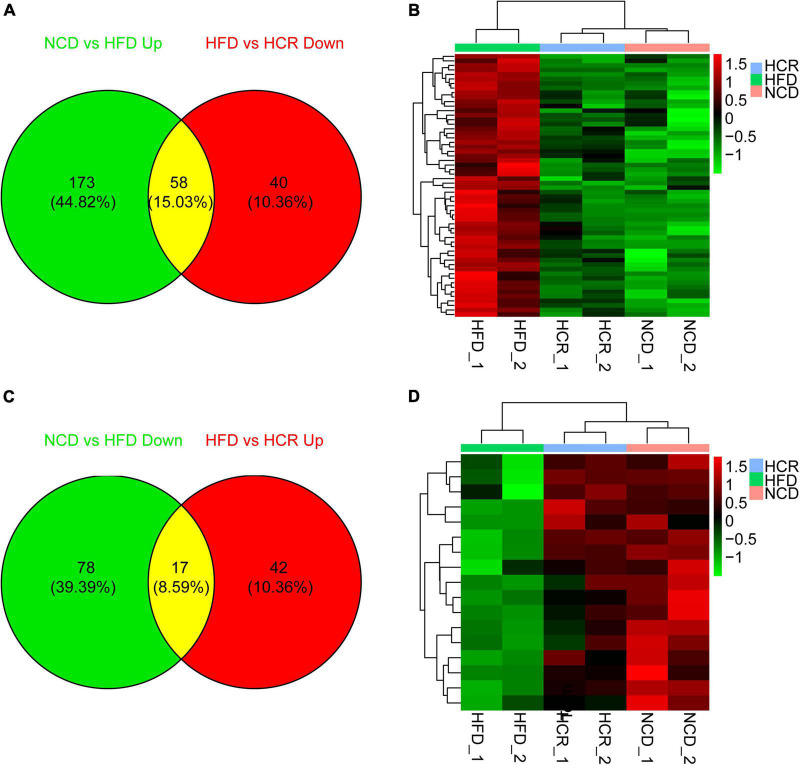
Cori changed hepatic gene expression profiles in HFD-fed mice. **(A)** 58 down-regulated differently expressed genes (DEGs) by treatment with Cori, and **(B)** dendrograms of the corresponding DEGs in indicated groups. **(C)** 17 up-regulated differently expressed genes (DEGs), and **(D)** dendrograms of the corresponding DEGs by treatment with Cori in indicated groups. Group are shown in columns and genes in rows. Gene expression is indicated as a color, with brighter green and red for lower and higher values, respectively.

To investigate the potential genes and mechanisms that were associated with hepatic lipid and glucose metabolism, the liver samples from NCD, HFD, and HCR-H (represented by HCR) mice were used for RNA-Seq, respectively. The raw reads for sequencing quality were general high with Q30 (%) ≥ 91.9%. Differentially expressed genes (DEGs) were defined based on three criteria: a fold change ≥ 2 (i.e., log 2 fold change > 1), *p* value < 0.05, and a mean expression (minimum) > 0.5 for at least one group. Using these parameters, we identified 326 DEGs (231 up-regulated and 95 down-regulated) in mice fed HFD compared to NCD group, whereas 157 DEGs (59 up-regulated and 98 down-regulated) were identified between HCR and HFD group (Table 2). On this basis, we further identified 75 overlapped DEGs (58 up-regulated, 17 down-regulated) in the livers between NCD vs. HFD group and HFD vs HCR group (the former group serves as control), as displayed by Venn diagram (Figures 4A,C). Furthermore, we performed hierarchical cluster analysis for DEGs among three groups and displayed distinctly different patterns of gene expression, indicated as dendrograms (Figures 4B,D).

**TABLE 2:** The summary of DEGs by RNA-seq.

Group comparison*	Up-regulated	Down-regulated	Total
NCD vs. HFD	231	95	326
HFD vs. HCR	59	98	157
NCD vs. HCR	87	49	136

*The former group serves as a reference for comparison.

It was worthy of note that the expression patterns between NCD and HCR group were more similar than those between NCD and HFD group by using the hierarchical cluster analysis(Figures 4B,D). In contrast, most of DEGs in NCD vs. HFD group had the opposite expression patterns to HFD vs. HCR group. E.g., *Cd36* and *Cyp7a1* were up-regulated in HFD mice, whereas down-regulated in HCR group; *Hsd3b5* and *Cspg5* were down-regulated in HFD mice, while up-regulated in HCR group. The down-regulated and up-regulated Cori-mediated DEGs (overlapped DEGs, NCD vs. HFD and HFD vs. HCR) were list in Tables 3, 4, respectively. Taken together, the Cori-dependent overlapped DEGs could serve as a featured gene expression signature of NAFLD, among which the potential genes could play pivotal roles for protecting from NAFLD and its associated metabolic disorders.

**TABLE 3:** The down-regulated DEGs in livers by treatment with Cori.

Gene symbol	Log2 fold change NCD vs. HFD	*P-value*	Log2 fold change HFD vs. HCR	*P-value*
Acss3	2.052	0.000	–1.605	0.000
Cyp2c38	2.053	0.000	–1.350	0.000
Cyp2a22	4.405	0.000	–3.252	0.000
Osbpl3	2.659	0.000	–1.968	0.000
Sema5b	2.746	0.000	–1.771	0.000
Gpc1	3.236	0.000	–1.334	0.000
Tpm2	2.429	0.000	–1.491	0.000
1810046K07Rik	1.989	0.001	–3.902	0.000
Arhgef9	1.751	0.000	–1.537	0.000
Cd36	1.559	0.000	–1.242	0.000
Slc22a27	6.141	0.000	–3.037	0.000
Tert	1.331	0.000	–1.176	0.000
Themis	5.742	0.000	–2.930	0.000
Cyp7a1	2.672	0.000	–1.471	0.000
Paqr7	1.480	0.000	–1.005	0.000
Cyp2b9	11.139	0.000	–2.707	0.000
Acot11	1.815	0.000	–1.513	0.000
Erbb4	1.527	0.000	–1.479	0.000
Gbp10	2.227	0.000	–1.242	0.000
Lrtm1	3.360	0.000	–2.648	0.000
H2-Q1	2.020	0.000	–1.090	0.001
Esrrg	1.300	0.000	–1.034	0.001
Aqp8	1.980	0.000	–1.120	0.001
Tmem237	1.309	0.025	–1.746	0.002
Thrsp	2.103	0.000	–1.312	0.003
Abcd2	1.178	0.022	–1.176	0.003
Chrm1	Inf	0.005	–Inf	0.004
Sytl4	1.771	0.000	–1.310	0.005
Hist1h4f	1.532	0.002	–1.324	0.005
Cfd*	Inf	0.000	–5.927	0.005
Pde6c	1.523	0.035	–1.991	0.006
Kbtbd11	1.223	0.050	–1.860	0.006
Eda	1.356	0.008	–1.336	0.007
Cyp17a1	1.699	0.013	–1.476	0.008
Stap1	2.199	0.000	–1.053	0.008
Lgals1	2.409	0.005	–2.159	0.009
Slc16a5	2.012	0.014	–1.961	0.009
Gm21885	2.019	0.004	–1.688	0.011
2900011O08Rik	3.279	0.027	–4.103	0.012
Pls1	1.573	0.037	–1.889	0.013
Slc22a29	4.599	0.000	–2.443	0.014
Pnldc1	1.925	0.002	–1.351	0.015
Acot4	1.300	0.015	–1.265	0.018
Cabp4*	Inf	0.000	–2.701	0.019
Hist1h4j	2.871	0.003	–2.005	0.019
Mas1*	Inf	0.001	–3.024	0.024
Zmat5	1.100	0.015	–1.011	0.025
Nupr1l	2.848	0.003	–1.862	0.026
Chek2	1.515	0.010	–1.190	0.028
Gna14	1.893	0.013	–1.243	0.028
Rpgrip1	2.549	0.003	–1.468	0.037
4931428L18Rik	2.242	0.005	–1.426	0.037
Lamb3	1.370	0.042	–1.373	0.037
Mt1	1.835	0.018	–1.563	0.041
Gstt3	1.305	0.046	–1.341	0.044
Aqp4	1.347	0.029	–1.198	0.045
Hhipl1	2.014	0.001	–1.005	0.047
Fitm1	1.811	0.008	–1.088	0.048

*Inf indicates the maximal value.

**TABLE 4:** The up-regulated DEGs in livers by treatment with Cori.

Gene symbol	Log2 fold change NCD vs HFD	*P-value*	Log2 fold change HFD vs. HCR	*P* value
Egfr	–1.691	0.000	1.513	0.000
Gstp1	–1.243	0.000	1.176	0.000
Cib3	–3.918	0.044	2.364	0.000
Hsd3b5	–1.447	0.003	1.133	0.000
Cspg5	–2.623	0.000	2.284	0.001
Carmil1	–1.014	0.000	1.335	0.001
Ugt2b38	–1.421	0.001	1.547	0.001
Dhrs9	–2.736	0.000	2.112	0.002
Cyp2c40	–3.789	0.000	2.170	0.002
Adgrf1	–3.564	0.002	1.548	0.003
Wnk4	–2.359	0.000	1.206	0.004
Cyp2c69	–4.060	0.000	2.872	0.009
Smad9	–1.774	0.018	1.103	0.016
Cd207	–1.938	0.000	1.210	0.032
Serpina9	–4.245	0.000	2.936	0.034
Shank1	–2.496	0.049	2.553	0.035
Esco2	–2.007	0.001	1.377	0.039

### Cori exerted obvious enrichment of GO terms and KEGG pathways for the overlapped DEGs

**FIGURE 5:**
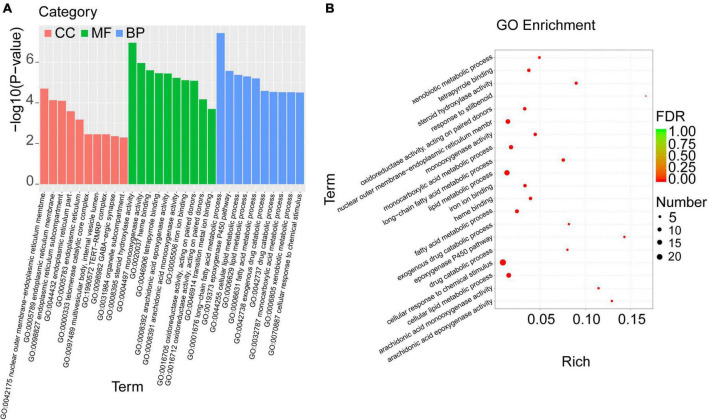
GO enrichment analysis was performed with the overlapped DEGs. **(A)** The categories in GO terms included cellular components (CC), molecular function (MF), and biological process (BP) were analyzed. **(B)** The top 20 significant GO terms of the overlapped DEGs in livers were chosen based on the order of *p* value from small to large.

To investigate the biological functions of the overlapped DEGs, the GO enrichment analysis was performed, and the categories in GO terms included cellular components (CC), molecular function (MF), and biological process (BP) were analyzed (Figure 5 and Table 5). As shown in Figure 5A, the major categories in cellular components (CC) were composed by nuclear outer membrane-endoplasmic reticulum membrane, endoplasmic reticulum membrane, endoplasmic reticulum part, endoplasmic reticulum, telomerase catalytic core complex, multivesicular body, internal vesicle lumen, TERT-RMPR complex, GABA-ergic synapse, organelle subcompartment. As for molecular function (MF), the overlapped DEGs were mainly enriched in steroid hydroxylase activity, monooxygenase activity, heme binding, tetrapyrrole binding, arachidonic acid epoxygenase activity, arachidonic acid epoxygenase monooxygenease activity, iron ion binding, oxidoreductase activity, acting on paired donors, and transition metal ion binding. Biological process (BP) analysis displayed that the critical genes were primarily enriched in long-chain fatty acid metabolic process, epoxygenase P450 pathway, cellular lipid metabolic process, lipid metabolic process, fatty acid metabolic process, exogenous drug catabolic process, monocarboxylic acid metabolic process, xenobiotic metabolic process, cellular response to chemical stimulus.

**TABLE 5:** Top 20 significant GO terms of the overlapped DEGs in livers.

Category	GO ID	Term	list	Total	*P-value*
BP	GO:0001676	long-chain fatty acid metabolic process	7	93	0.000
MF	GO:0008395	steroid hydroxylase activity	6	67	0.000
MF	GO:0004497	monooxygenase activity	7	158	0.000
MF	GO:0020037	heme binding	7	179	0.000
BP	GO:0019373	epoxygenase P450 pathway	4	28	0.000
MF	GO:0046906	tetrapyrrole binding	7	188	0.000
MF	GO:0008392	arachidonic acid epoxygenase activity	4	31	0.000
BP	GO:0044255	cellular lipid metabolic process	14	940	0.000
BP	GO:0006629	lipid metabolic process	16	1242	0.000
MF	GO:0008391	arachidonic acid monooxygenase activity	4	35	0.000
BP	GO:0006631	fatty acid metabolic process	9	374	0.000
MF	GO:0005506	iron ion binding	7	212	0.000
MF	GO:0016705	oxidoreductase activity, acting on paired donors, with incorporation or reduction of molecular oxygen	7	214	0.000
CC	GO:0042175	nuclear outer membrane-endoplasmic reticulum membrane network	13	930	0.000
BP	GO:0042738	exogenous drug catabolic process	4	49	0.000
BP	GO:0042737	drug catabolic process	4	50	0.000
BP	GO:0032787	monocarboxylic acid metabolic process	10	571	0.000
BP	GO:0006805	xenobiotic metabolic process	5	102	0.000
BP	GO:0070887	cellular response to chemical stimulus	24	2902	0.000
BP	GO:0035634	response to stilbenoid	3	18	0.0000

Furthermore, the top 20 significant GO terms of the overlapped DEGs in livers were chosen based on the order of p value from small to large, and displayed in Figure 5B and Table 5. It was found that the overlapped DEGs were dominantly enriched in long-chain fatty acid metabolic process, steroid hydroxylase activity, steroid hydroxylase activity, monooxygenase activity, heme binding, epoxygenase P450 pathway, tetrapyrrole binding, arachidonic acid epoxygenase activity, cellular lipid metabolic process, lipid metabolic process, arachidonic acid monooxygenase activity, fatty acid metabolic process, etc. These functions were closely correlated with the physiological regulatory roles in liver metabolism, especially the metabolic homeostasis of lipids and cholesterols.

**FIGURE 6:**
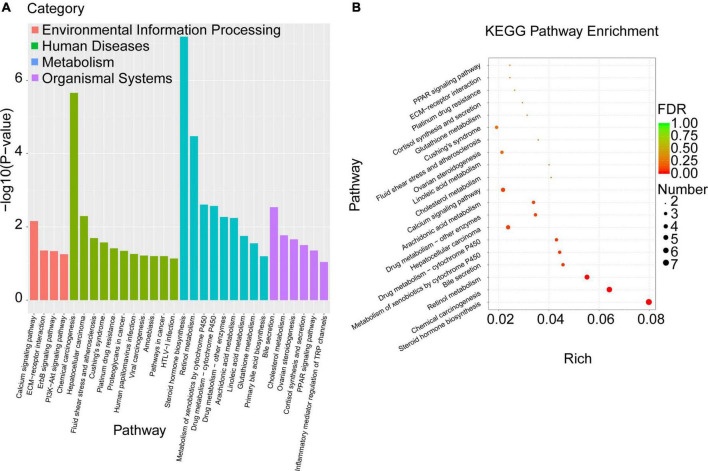
KEGG pathway enrichment analysis was performed with the overlapped DEGs. **(A)** The enriched KEGG pathways were clustered into four main categories, including environmental information processing, human diseases, metabolism, and organismal systems, which were further divided into several subcategories. **(B)** The top 20 significant KEGG pathways of the overlapped DEGs in liver were selected based on the order of *p* value from small to large.

To explore the biological pathways of the overlapped DEGs, the KEGG enrichment analysis were subsequently conducted. As shown in Figure 6A and Table 6, the enriched KEGG pathways were generally clustered into four categories, including environmental information processing, human diseases, metabolism, and organismal systems, which were further divided into several subcategories, respectively. As for environmental information processing, the key genes were mainly enriched in calcium signaling pathway, ECM-receptor interaction, ErbB signaling pathway, PI3k-Akt signaling pathway. As for Human diseases, the key genes were mainly enriched in chemical carcinogenesis, hepatocellular carcinoma, fluid shear stress and atherosclerosis, Cushing’ syndrome, platinum drug resistance, proteoglycans in cancer, human papillomavirus infection, viral carcinogenesis, amebiasis, pathways in cancer, HTLV-I infection. As for metabolism, the key genes were mainly enriched in steroid hormone biosynthesis, retinol metabolism, metabolism of xenobiotics by cytochrome P450, drug metabolism- cytochrome P450 or other enzymes, arachidonic acid metabolism, linoleic acid metabolism, glutathione metabolism, primary bile acid biosynthesis. As for organismal systems, the key genes were mainly enriched in bile secretion, cholesterol metabolism, ovarian steroidogenesis, cortisol synthesis and secretion, PPAR signaling pathway, inflammatory mediator regulation of TRP channels.

**TABLE 6:** Top 20 significant enriched pathways of the overlapped DEGs in livers.

Category	Pathway ID	*P* value	Genes
Steroid hormone biosynthesis	mmu00140	0.000	Hsd3b5/Cyp17a1/Cyp2b9/Ugt2b38/Cyp7a1/Cyp2c38/Cyp2c40
Chemical carcinogenesis	mmu05204	0.000	Gstt3/Gstp1/Cyp2b9/Ugt2b38/Cyp2c38/Cyp2c40
Retinol metabolism	mmu00830	0.000	Cyp2b9/Ugt2b38/Dhrs9/Cyp2c38/Cyp2c40
Metabolism of xenobiotics by cytochrome P450	mmu00980	0.002	Gstt3/Gstp1/Ugt2b38
Drug metabolism - cytochrome P450	mmu00982	0.003	Gstt3/Gstp1/Ugt2b38
Bile secretion	mmu04976	0.003	Aqp4/Aqp8/Cyp7a1
Hepatocellular carcinoma	mmu05225	0.005	Tert/Gstt3/Gstp1/Egfr
Drug metabolism - other enzymes	mmu00983	0.005	Gstt3/Gstp1/Ugt2b38
Arachidonic acid metabolism	mmu00590	0.006	Cyp2b9/Cyp2c38/Cyp2c40
Calcium signaling pathway	mmu04020	0.007	Gna14/Chrm1/Egfr/Erbb4
Cholesterol metabolism	mmu04979	0.017	Cd36/Cyp7a1
Linoleic acid metabolism	mmu00591	0.018	Cyp2c38/Cyp2c40
Fluid shear stress and atherosclerosis	mmu05418	0.020	Gstt3/Gstp1/Gpc1
Ovarian steroidogenesis	mmu04913	0.022	Hsd3b5/Cyp17a1
Cushing’s syndrome	mmu04934	0.027	Hsd3b5/Cyp17a1/Egfr
Glutathione metabolism	mmu00480	0.028	Gstt3/Gstp1
Cortisol synthesis and secretion	mmu04927	0.031	Hsd3b5/Cyp17a1
Platinum drug resistance	mmu01524	0.038	Gstt3/Gstp1
ECM-receptor interaction	mmu04512	0.044	Cd36/Lamb3
PPAR signaling pathway	mmu03320	0.044	Cd36/Cyp7a1

Moreover, the top 20 significant KEGG pathways of the overlapped DEGs in liver were selected based on the order of p value from small to large, and displayed in Figure 6B and Table 6. It was found that overlapped DEGs were mainly enriched in steroid hormone biosynthesis, chemical carcinogenesis, retinol metabolism, bile secretion, metabolism of xenobiotics by cytochrome P450, drug metabolism-cytochrome P450, cholesterol metabolism, linoleic acid metabolism, arachidonic acid metabolism, etc. These metabolic pathways are tightly associated with liver metabolic balance, supporting the protective effects of Cori for the improvement of NAFLD.

### Cori-dependent PPI network for the overlapped DEGs and hub proteins against NAFLD

**FIGURE 7:**
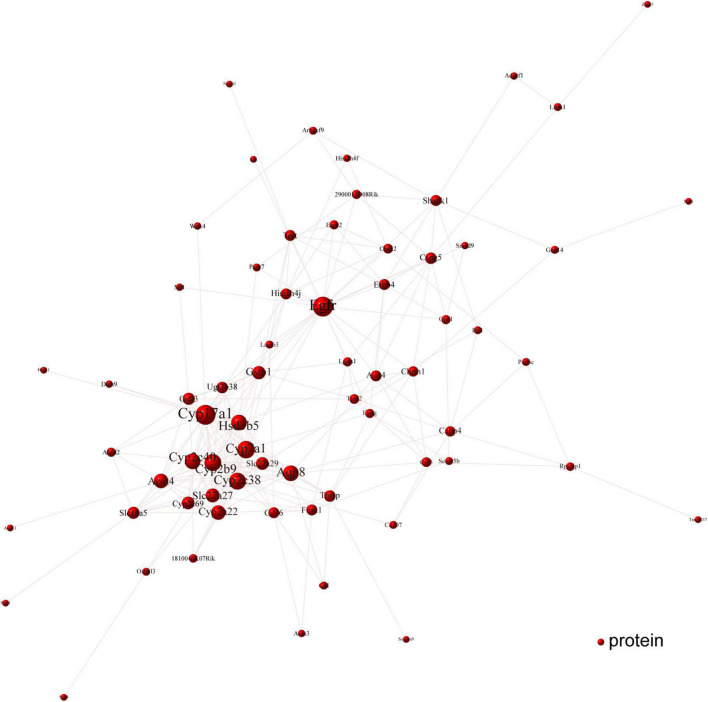
PPI network analysis was performed with the overlapped DEGs. The connections between nodes indicate the interactions between the proteins encoded by the overlapped DEGs (red nodes). The visualization for Cori-dependent hub proteins were selected with a PPI score over 0.9, such as *Cyp7a1*, *Hsd3b5*, *Cyp17a1*, *Cyp2c38*, *Cyp2b9*, *Egfr*, *Erbb4*, *Cyp2c40*, *Gna14*, *Chrm1*, *Cspg5*, and *Gpc1*.

To determine the Cori-mediated hub proteins (namely putative gargets) that may play protective roles against NAFLD, a visualized PPI network of the overlapped DEGs was constructed by Cytoscape software according to STRING database. As shown in Figure 7, the connections between nodes (referred to proteins) in the PPI analysis were tightly established and visualized, which indicate the interactions between the proteins encoded by the overlapped DEGs. The proteins with more connections located in the central node, which may play important roles in the regulation of NAFLD, and were selected as the hub proteins. Consequently, the visualization for Cori-mediated hub proteins (with PPI score > 0.9), in the analysis of STRING, were *Cyp7a1*, *Hsd3b5*, *Cyp17a1*, *Cyp2c38*, *Cyp2b9*, *Egfr*, *Erbb4*, *Cyp2c40*, *Gna14*, *Chrm1*, *Cspg5*, and *Gpc1* (Figure 7).

**FIGURE 8:**
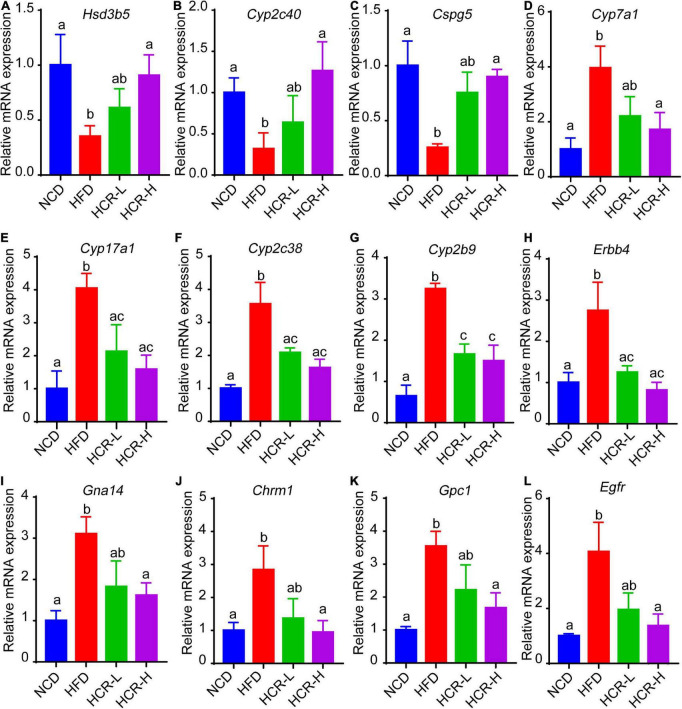
Relative mRNA expression of hub genes via qPCR analysis. Compared with HFD group, The mRNA expression of *Hsd3b5*, *Cyp2c40*, *Cspg5* in HCR group were increased; by contrast, the mRNA expression of *Cyp7a1*, *Cpy17a1*, *Cyp2c38*, *Cyp2b9*, *Egfr*, *Erbb4*, *Gna14*, *Chrm1*, and *Gpc1* in HCR group were decreased.

Next, we further verified the mRNA expression of the hub proteins by using qPCR assays. As shown in Figure 8, Cori treatment could effectively increase the mRNA expression levels of *Hsd3b5*, *Cyp2c40*, *Cspg5*; whereas decrease the expression of *Cyp7a1, Cpy17a1*, *Cyp2c38*, *Cyp2b9*, *Egfr*, *Erbb4*, *Gna14*, *Chrm1*, and *Gpc1*. Additionally, we also analyzed the mRNA expression levels of several key factors that closely associated with lipid and cholesterol metabolism, although their PPI score less than 0.9. For example, *Cd36*, a fatty acid translocase, promotes the uptake of FFAs and cholesterol under HFD conditions, which may contribute to the pathogenesis of NAFLD and its related metabolic disorders (24, 25). Consistent with the RNA-Seq results and previous reports, we demonstrated that the hepatic CD36 expression was obviously induced in HFD-fed mice compared to NCD mice, whereas Cori treatment dramatically reversed its expression to a level similar to NCD group (Supplementary Figure 1F). Taken together, these results suggests that the overlapped DEGs aforementioned, especially the hub proteins, may play a key role on the Cori-mediated improvement of NAFLD.

## Discussion

In this study, we focused on the protective roles of Cori against the progression of NAFLD in mice under high fat diet feeding conditions. We found that Cori treatment significantly attenuated HFD- induced high NAFLD activity score (NAS) and liver injury. Moreover, by treatment with Cori, the hepatic lipid deposition and plasma lipid concentrations were remarkably improved in mice fed with high fat diet. Cori administration ameliorated NAFLD associated metabolic disorders such as glucose intolerance and insulin resistance in HFD-fed mice. More importantly, applying RNA-Seq analysis, we noticed that Cori treatment could dramatically change HFD-induced gene expression profiles in liver. Furthermore, by analysis of the overlapped differentially expressed genes (DEGs) obtained from NCD vs HFD group and HFD vs HCR group, we observed a marked enrichment of GO terms and KEGG pathways, which were closely associated with lipid metabolism and glucose homeostasis. Thus, the novel findings achieved in transcriptome provided valuable information for Cori deterrence of NAFLD. Particularly, PPI network analysis revealed the Cori-mediated potential hub proteins against NAFLD. Additionally, Cori-mediated overlapped DEGs could serve as a featured NAFLD-associated gene expression signature, and thus be used in diagnosis, treatment, as well as drug discovery and development for fatty liver disease and metabolic disorders.

We firstly constructed the NAFLD model in mice by feeding a high fat diet. NAFLD activity score (NAS) is a wide-accepted and standardized approach to histologically evaluate the severity of NAFLD, which indicates the general pathology scores for hepatic steatosis, inflammation, and ballooning degeneration (21). In our results, HFD-fed mice displayed an obvious increase in liver weight (and liver index), and developed severe hepatic steatosis along with high NAFLD activity score (NAS), which together indicated that we successfully established a NAFLD model. By treating mice with Cori, we observed an obvious improvement in phenotypic characteristics of NAFLD under HFD conditions in a dose-dependent manner, including reduced number and size of lipid droplets, and alleviated NAFLD activity score (NAS). It is known that elevated plasma ALT and AST levels are two common biomakers of hepatocellular injury (26). In this study, Cori significantly rescued HFD-induced increase of ALT and AST contents in plasma, clearly indicating its protective roles on liver function in the NAFLD progression.

**FIGURE 9:**
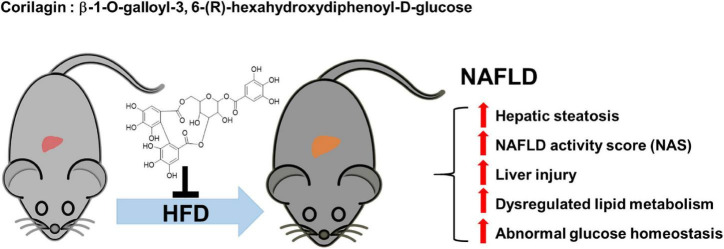
The protective roles of Corilagin against NAFLD development in HFD-fed mice. HFD feeding induced NAFLD, which was accompanied by hepatic steatosis, high NAFLD activity score (NAS), liver injury, abnormal lipid metabolism and glucose homeostasis; however, Cori treatment significantly attenuated the progress of NAFLD.

We next assumed the protective roles of Cori in the regulating NAFLD-associated metabolic disorders of lipid and glucose. As a crucial metabolic organ, the liver regulates numerous physiological processes, such as the stability of lipid metabolism and glucose homeostasis between liver and blood (27). In the case of hypernutrition, hepatocellular lipotoxic injury mainly attributes to the abnormal metabolism of lipids such free fatty acids (FFAs) and cholesterols, and the injured steatotic hepatocyte may further accelerate the progression of NAFLD via a self-perpetuating vicious cycle (11). As expected, NAFLD model mice showed a remarkable increase in hepatic TG and TC contents, whereas Cori treatment blocked these effects, and ameliorated HFD-induced fatty liver. Also, hyperlipidemia is closely related to the pathological development of NAFLD. In our results, administration of Cori to HFD-fed mice led to an improved plasma lipid profiles mediated by NAFLD, as evidenced by decreased TG, TC and LDL-c levels, as well as elevated HDL-c content. Consistent with these, we observed a marked reduction of epididymal fat weight as well as epididymal fat index (%) in HFD-fed mice when their response to Cori, which might contribute to the decreased levels of FFAs and FABP4 in plasma. On the other hand, HFD-induced glucose intolerance and insulin resistance are pivotal factors, which are associated with the disorders of lipid metabolism, and may further accelerate the progression of NAFLD. In this study, Cori treatment attenuated HFD-induced increase of fasting blood glucose and insulin levels, and strongly reversed the HOMA-IR value, an index of systemic insulin resistance (28). Specifically, Cori- treated mice under HFD exhibited an obvious improvement of glucose tolerance and insulin sensitivity, as evidenced by GTT and ITT assays. Collectively, Cori supplement could play protective roles against hepatic steatosis, high NAFLD activity score (NAS), as well as liver injury in HFD-induced NAFLD mice, which might not only be involved in alleviating HFD-induced lipid metabolism disorders, but also be involved in ameliorating HFD-induced glucose intolerance and insulin resistance (Figure 9).

Further, we employed the RNA-Seq to explore the underlying genes and mechanisms that were associated the hepatic lipid and glucose metabolism. Interestingly, we observed an obvious altered gene expression profiles and identified 157 DEGs (59 up-regulated and 98 down-regulated) in mice with HFD feeding by intervention with Cori. Among these, we further identified 75 overlapped DEGs (58 up-regulated and 17 down-regulated) in the livers between NCD vs HFD group and HFD vs HCR group. Of note, we found that the liver samples of NCD and HCR group have more similar expression patterns than NCD and HFD group does, as depicted by dendrograms of hierarchical cluster analysis of the overlapped DEGs. Therefore, the overlapped DEGs might be considered as a NAFLD-related gene expression signature, which would promote research on molecular diagnosis, treatment, and drug development of NAFLD.

We next explored the biological functions and metabolic pathways of the overlapped DEGs by using GO and KEGG enrichment analyses, respectively. In our results, the top 20 significant GO terms of the overlapped DEGs were closely associated with metabolic balance, especially lipid and cholesterol metabolic homeostasis. These results further support results aforementioned that Cori treatment attenuates HFD-induced liver steatosis and high NAFLD activity score (NAS), which might be involved in rebuilding the balance of lipid homeostasis, and thereby blocks NAFLD development in HFD mice. Interestingly, we noticed that the bile biosynsthesis-related biological processes were largely enriched, as determined in the catergory of molecular function, suggesting that Cori might restored the dysregulated BA metabolisms mainly caused by hepatic lipid accumulation in livers of mice with HFD feeding (29). In our study, we did not detect bile acid content in each group, further studies regarding the role of Cori in regulating the conversion of cholesterol catabolism to bile acids need to be clarified. Consistent with the GO analysis, the top 20 significant KEGG pathways of the overlapped DEGs were mainly enriched in steroid hormone biosynthesis, lipid and cholesterol metabolism, bile secretion and retinol metabolism, etc. In particular, after clustered into indicated categories, the biological pathways were primarily enriched in lipid and cholesterol metabolism, bile biosynthesis and secretin, cortisol synthesis and secretion, hepatocellular carcinoma, PPAR signaling pathway, PI3K-AKT signaling pathway, etc. These results suggest that Cori might ameliorate HFD-induced NAFLD by the regulating these biological processes and metabolic pathways, which were closely associated with the hepatic lipid metabolism and glucose homeostasis.

We also conducted a visualized PPI network using the overlapped DEGs, which facilitated to discover the Cori-dependent hub proteins (namely putative targets) in livers regulating NAFLD. In our results, several proteins, in the analysis of STRING database, were selected as hub proteins (PPI score > 0.9), and their expression were subsequently verified by qPCR assays. These hub proteins may play pivotal roles in Cori-mediated improvement of NAFLD. For example, *Hsd3b5*, one was involved in steroid hormone metabolism, was negatively correlated with the severe grade of hepatic steatosis (30, 31). Consistent with this, Cori rescued the decreased *Hsd3b5* expression in HFD-fed mice, which might be likely involved in its protective role against NAFLD development. Additionally, some differentially expressed proteins, e.g. *Cd36*, the PPI score of which does not met with our criteria (i.e., PPI > 0.9) for hub proteins, may still play a key role in regulating the progress of NAFLD. It has been reported that elevated hepatic *Cd36* expression was closely associated with increased hepatic and plasma triglyceride contents in both humans and rodents, which could contribute to the progression of NAFLD (32, 33). In this study, the elevated hepatic *Cd36* expression observed in mice fed a HFD was effectively suppressed by treatment with Cori, which was recovered to a level similar to NCD group. Therefore, we speculated that the decreased expression of *Cd36* in liver might, at least in part, play a role in improving hepatic and plasma lipid contents under HFD conditions. Collectively, we developed a new characteristic gene expression signature related to NAFLD by applying the Cori-dependent DEGs aforementioned, especially the hub proteins in the analysis of PPI network, which thus would facilitates the diagnosis, treatment, as well as drug discovery and development for NAFLD in the near future.

## Conclusion

In our results, Cori treatment exhibited beneficial protective effects against HFD-induced hepatic steatosis, high NAFLD activity score (NAD) and liver injury. Moreover, Cori ameliorated NAFLD associated metabolic disorders such as glucose intolerance and insulin resistance in HFD-fed mice. Furthermore, Cori dramatically changed HFD-induced liver gene expression profiles, and produced an overlapped differentially expressed genes (DEGs) ——Cori-dependent DEGs. The Cori-dependent DEGs were primarily enriched in GO terms and KEGG pathways associated with lipid and glucose metabolism. The novel findings achieved in transcriptome provided valuable information for Cori deterrence of NAFLD. Additionally, the Cori-dependent DEGs, especially the hub proteins in the analysis of PPI network, can serve as a featured NAFLD-associated gene expression signature for the diagnosis, treatment, as well as drug discovery and development of NAFLD in the near future.

## Data availability statement

The data presented in this study are deposited in the Sequence Read Archive (SRA) repository, accession number PRJNA862909.

## Ethics statement

The animal study was reviewed and approved by the ethics committee of Shanghai Jiao Tong University School of Medicine, Shanghai, China.

## Author contributions

YW and GW: conceptualization and resources. ML, RZ, YW, and GW: methodology and data curation. ML, RZ, YW, HG, KZ, JW, CZ, YJ, and ZM: investigation. ML, RZ, YW, ZM, XC, and GW: original draft preparation and review and editing, HS, XC, ML, and GW: visualization and funding. All authors contributed to the article and approved the submitted version.
