# Supplementary material for: Corilagin prevents non-alcoholic fatty liver disease via improving lipid metabolism and glucose homeostasis in high fat diet-fed mice
Source: Front Nutr. 2022 Aug 17;9:983450. doi: 10.3389/fnut.2022.983450 (PMC9443665; doi:10.3389/fnut.2022.983450)
Supplement: Supplementary file 1 [file Data_Sheet_1.PDF]

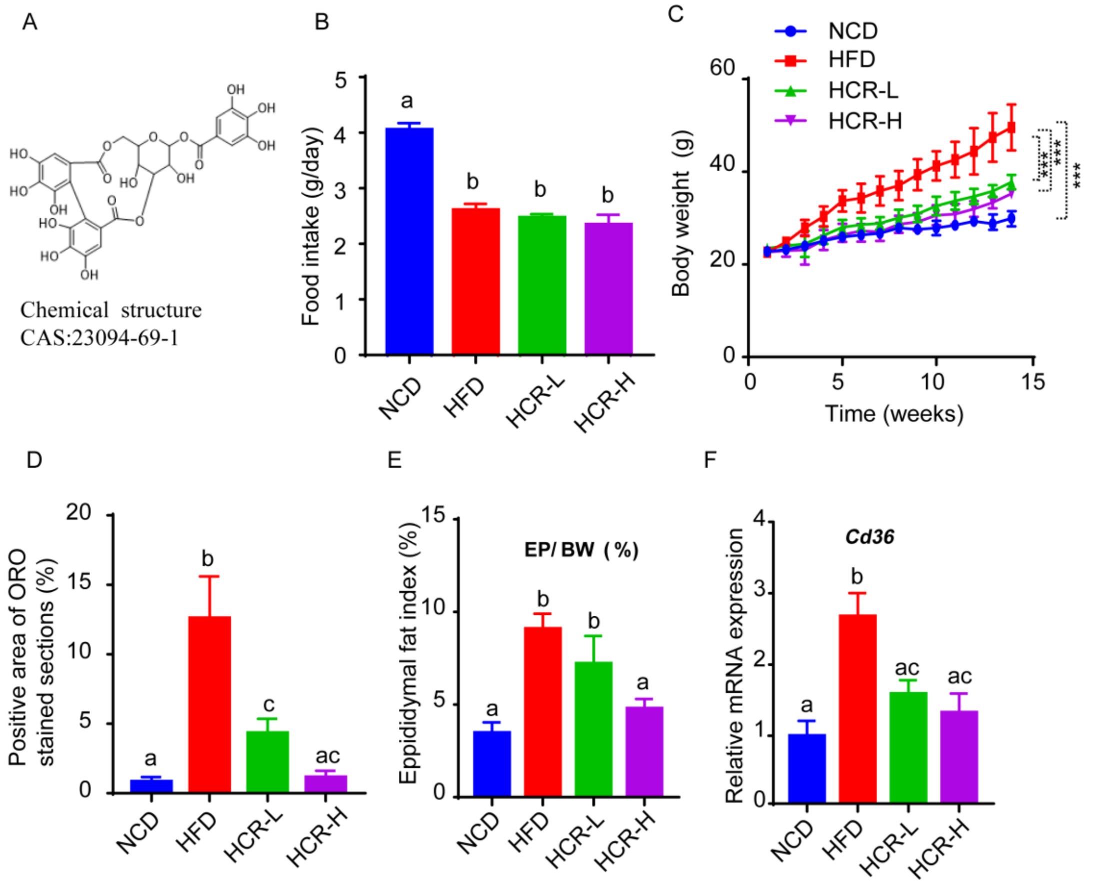

Supplementary Figure 1. The parameters alterations in mice-fed HFD after treatment with corilagin (Cori). The chemical structure and the CAS number of sorafenib and sunitinib, respectively. (A) The chemical structure and the CAS No. for Cori. (B) Food intake, (B) Body weight changes, (C) Positive area of Oil red O Staining (%), (E) eppididymal fat index (%), and (F) Relative mRNA expression in indicated groups. Values were means ± SD, and for statistical analysis, one-way (B, D-F) or two way (C) ANOVA were performed between indicated groups.
